# The pharmacological mechanism of chaihu-jia-longgu-muli-tang for treating depression: integrated meta-analysis and network pharmacology analysis

**DOI:** 10.3389/fphar.2023.1257617

**Published:** 2023-09-21

**Authors:** Yang Zhao, Dan Xu, Jing Wang, Dandan Zhou, Anlan Liu, Yingying Sun, Yuan Yuan, Jianxiang Li, Weifeng Guo

**Affiliations:** ^1^ First Clinical Medical College, Nanjing University of Chinese Medicine, Nanjing, China; ^2^ Taicang TCM Hospital Affiliated to Nanjing University of Chinese Medicine, Taicang, China; ^3^ Taicang Hospital of Traditional Chinese Medicine, Taicang, China; ^4^ Department of Respiratory and Critical Care Medicine, Jiangsu Province Hospital of Chinese Medicine, Affiliated Hospital of Nanjing University of Chinese Medicine, Nanjing, China; ^5^ School of Chinese Medicine School of Integrated Chinese and Western Medicine, Nanjing University of Chinese Medicine, Nanjing, China

**Keywords:** depression, chaihu-jia-longgu-muli-tang, meta-analysis, network pharmacology, inflammation

## Abstract

**Aim:** Chaihu-jia-Longgu-Muli-tang (CLM) is derived from “Shang Han Lun” and is traditionally prescribed for treating depression. However, there is still a lack of evidence for its antidepressant effects, and the underlying mechanism is also unclear. This study aimed to assess clinical evidence on the efficacy of CLM in patients with depression using a meta-analysis and to explore its underlying antidepressant molecular mechanisms via network pharmacology.

**Methods:** Eight open databases were searched for randomized controlled trials (RCTs) comparing the effects of CLM alone or combined with serotonin-norepinephrine reuptake inhibitors (SNRIs) and selective serotonin reuptake inhibitors (SSRIs) in patients with depression, evaluating the total effective rate of the treatment group (CLM alone or combined with SSRIs/SNRIs) and the control group (SNRIs or SSRIs), and comparing changes in depression scale, anxiety scale, sleep scale, inflammation indicators and adverse effects. Subsequently, the active ingredients and target genes of CLM were screened through six databases. Then Gene Ontology (GO) and Kyoto Encyclopedia of Genes and Genomes (KEGG) analysis and protein-protein interaction (PPI) network and topology analysis were performed. Finally, Molecular docking was applied to evaluate the binding affinity between components and predicted targets.

**Results:** Twenty-four RCTs with a total of 2,382 patients were included. For the efficacy of antidepression and adverse effects, whether CLM alone or in combination with SSRIs/SNRIs, the treatment group has no inferior to that of the control group. Additionally, the intervention of CLM + SSRI significantly improved the symptoms of anxiety and insomnia, and reduced serum IL-6 and TNF-α levels. For network pharmacology, a total of 129 compounds and 416 intersection targets in CLM were retrieved. The interaction pathway between CLM and depression is mainly enriched in PI3K-Akt, JAK-STAT, and NF-κB signaling pathway, PIK3R1, MAPK3, and AKT1 may be the potential targets of Stigmasterol, β-stiosterol, coumestrol.

**Conclusion:** Compared to SSRIs/SNRIs alone, CLM is more effective and safe in treating depression. It not only significantly alleviates depressive mood, but improves symptoms such as anxiety and insomnia, with fewer side effects, especially in combination with SSRI. Its antidepressant mechanism may be correlated with the regulation of the PI3K/Akt signaling pathway and inhibiting inflammatory response.

## 1 Introduction

Depression is a debilitating neuropsychological disorder that impairs daily functioning and is characterized by a constant sense of melancholy, loss of interest, and intellectual disability. Globally, major depression disorder (MDD) and dysthymia are responsible for approximately 46.9 million disabilities annually, with over 800,000 suicidal deaths mostly accompanied by neurological and psychiatric disorders such as MDD (Li et al., 2022a; Moitra et al., 2022). The number of patients with depression reached 542 million in 2015, with an 18.4% global increase between 2005 and 2015 (Disease and Prevalence, 2016). Due to the effects of coronavirus disease 2019, the number of patients with depression worldwide increased by 27.6% and depression is estimated to become the leading cause of death by 2030 (COVID-19 Mental Disorders Collaborators, 2021). Therefore, discovering efficient and safe treatment modalities is currently highly needed. There are three main interventions for treating depression that are currently used: 1) pharmaceutical antidepressants, such as SNRIs and SSRIs, among others; 2) research-based psychotherapy, such as cognitive behavioral therapy and interpersonal psychotherapy; and 3) physical therapy, such as vagus nerve stimulation or repeated transcranial magnetic stimulation. However, about one-third of patients still do not achieve remission after four consecutive antidepressant trials, and at least 50%–80% of patients still exist recurrence, with a progressive increase in severity and frequency, this mainly attributed to increased resistance in depression (Borbely et al., 2022). Furthermore, patients’ resistance to antidepressants is often accompanied by high levels of serum inflammatory factors such as interleukin-6 (IL-6) and tumor necrosis factor-α (TNF-α) (Beurel et al., 2020). Heightened inflammation can also exacerbate mood disorders such as anxiety and anhedonia, as well as somatic symptoms such as insomnia and gastrointestinal disorders (Kiecolt-Glaser et al., 2015). Thus, it is still necessary to find or develop antidepressant treatments with high-efficiency and fewer side effects.

Traditional Chinese Medicine (TCM) operates on the understanding that the human body is an organic whole, and the viscera coordinate with each other in function and influence each other in pathology (Xiang-yu et al., 2021). The pathogenesis of depression as viewed in TCM is a disorder of the Shao Yang pivot and dysfunction of Yin and Yang. Shao Yang is the pivot for the rise and fall of the Qi movement; Shao Yang dysfunction leads to depression and insomnia. When the Shao Yang channel restrains the Qi, the body will feel cold and physically heavy and patients will show appetite loss (Hanying et al., 2022). CLM is a TCM prescription derived from “Shang Han Lun” that is prescribed to treat mental diseases caused by the imbalance of the pivot of Shao Yang and the loss of Yin and Yang. It has the effects of mediating the Shao Yang pivot and tranquilizing the mind (Wan et al., 2021). Numerous studies have shown that CLM contributes to improvements in dementia, insomnia, anxiety, and depression (Yuanwen et al., 2022). Although the efficacy and safety of CLM for the treatment of depression have been evaluated in many studies, determining a reliable basis for evidence-based medicine is difficult due to deviations in the included studies, literature updates, and inconsistencies in patient grouping and comorbidities (Li et al., 2018; Wan et al., 2021). The potential mechanism behind CLM’s antidepressant effect may be related to the inhibition of N-methyl-D-aspartate (NMDA) receptors to increase hippocampal synaptic plasticity, positively regulation of neuronal apoptosis, and inhibition of neuroinflammation (Liu et al., 2010; Wang et al., 2018; Lizhi et al., 2021). However, owing to the intricacy of the compound constituents, particularly the molecular target mechanisms of its active substances, the mechanisms associated with its antidepressant effect remain unclear. As a novel discipline, network pharmacology can effectively and systematically explore correlations between traditional Chinese medicine substances, targets, and diseases by constructing a variety of network models comprehensively using multiple platforms and technologies to help explore potential mechanisms (Chen et al., 2018).

Based on the above background, CLM has a wide range of antidepressant efficacy, but with still a lack of clinical evidence, and its antidepressant mechanism is still unclear due to the complexity of TCM compound ingredients. Therefore, this study aimed to assess clinical evidence on the efficacy of CLM in patients with depression using a meta-analysis and to explore its underlying antidepressant molecular mechanisms via network pharmacology.

## 2 Materials and methods

### 2.1 Meta-analysis

#### 2.1.1 Data sources and searches

**FIGURE 1:**
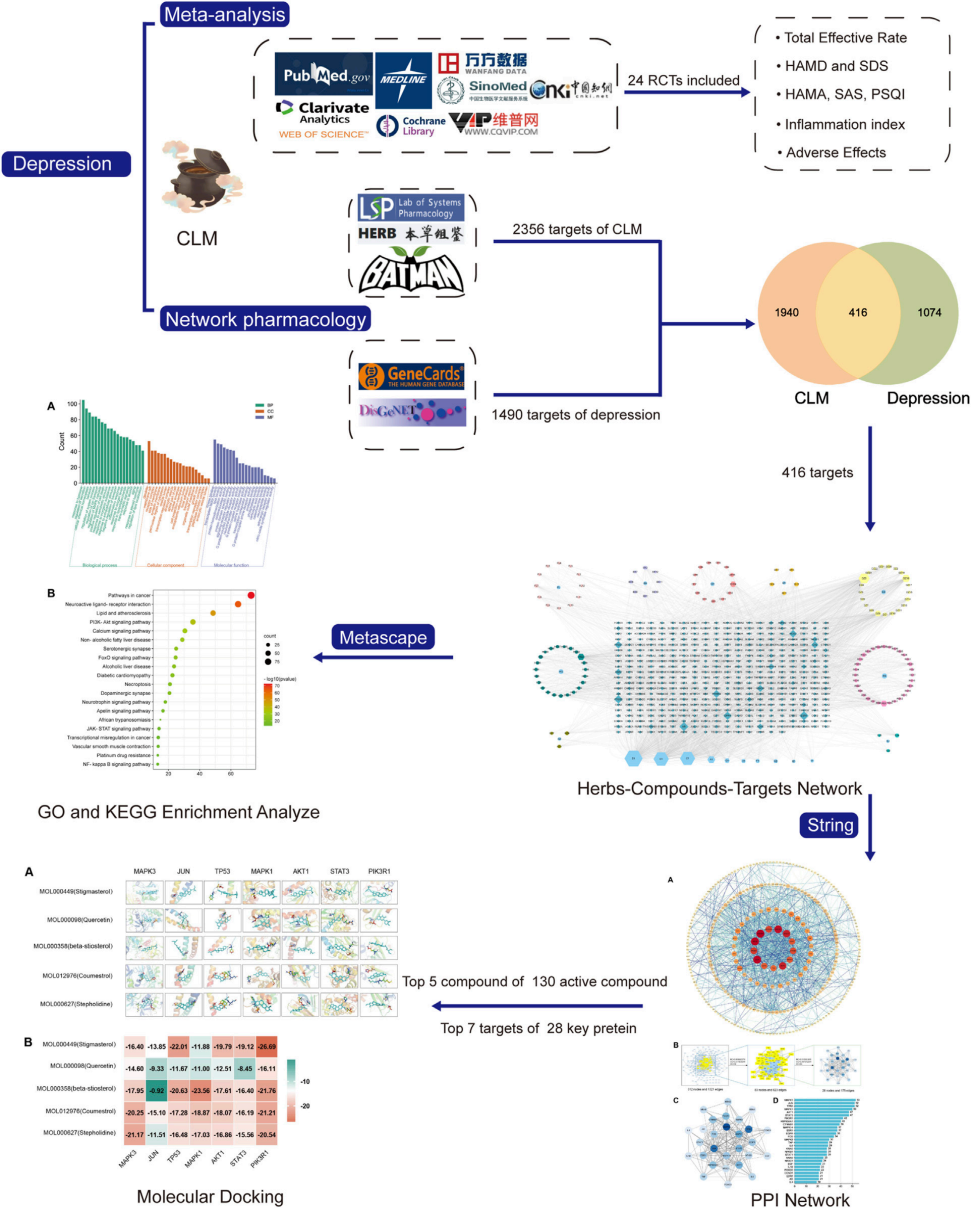
Flow chart of the investigation of CLM in the treatment of depression.

Clinical trials were searched in the PubMed, Web of Science, MEDLINE, Cochrane Library, China National Knowledge Internet, VIP, Wanfang, and SinoMed databases from the inception of each database until December 2022. This meta-analysis complied with the Preferred Reporting Items for Systematic Reviews and Meta-Analyses (Page et al., 2021) (protocol shown in Figure 1). The search strategy is shown in Supplementary Table S1.

#### 2.1.2 Inclusion and exclusion criteria

Due to the limited exact treatment methods for depression at present, physical therapy has the disadvantage of large side effects and poor compliance (e.g., repeated transcranial magnetic stimulation), and uneven efficacy (e.g., Acupuncture, massage, music, etc.). Although mainstream antidepressants (e.g., SSRIs and SNRIs) have exact therapeutic effects and are widely accepted, there are still some limitations we mentioned above, so we have made the following provisions in the inclusion and exclusion criteria.

The inclusion criteria were as follows: 1) randomized controlled trials retrieval was limited to published in Chinese and English; 2) studies including participants clearly diagnosed with major depression disorder according to the Chinese Classification of Mental Disorders Third Edition (CCMD-3) or the Diagnostic and Statistical Manual of Mental Disorders (DSM-IV) (Regier et al., 2013); 3) Studies including at least one depression related outcome indicator such as Hamilton Anxiety Rating Scale (HAMD) or Self-rating Depression Scale (SDS); 4)the treatment group received CLM or a combination of CLM and SSRIs or SNRIs, the control group treated SSRIs/SNRIs alone. In addition, the intervention of CLM was added or subtracted according to syndrome differentiation, and the botanical drugs mainly including Radix Bupleuri [Chai Hu], Fossilia Ossia Mastodi [Longgu], Radix Scutellariae [Huang Qin], Zingiber rhizoma [Shengjiang], Ginseng radix [Renshen], Cinnamomi cortex [Guizhi], Hoelen [Fuling], Pinelliae Tuber [Banxia], Radix et Rhizoma Rhei [Dahuang], Ostreae Testa [Muli], and Zizyphi fructus [Dazao]. The composition of CLM is shown in Supplementary Table S2.

The exclusion criteria were as follows: 1) incomplete data; 2) inclusion of drug-induced secondary depression, somatic or psychiatric disease-induced secondary depression, *postpartum* depression, or menopausal depression; 3) other comorbid conditions, 4) interventions combining other traditional Chinese medicine formulas or physical therapy (e.g., acupuncture, massage, music, etc.).

#### 2.1.3 Data extraction and quality assessment

The search terms and their common synonyms in each database were as follows: “Depression OR Depressive” AND “chaihu jia longgu muli decoction OR CLM.” All retrieved studies were imported into Endnote20 software for screening and eliminating duplicates according to abstracts. Two researchers (YS. and YY.) extracted the information trial characteristics (study, sample size, gender, average age, interventions, duration, average course, and outcomes (Total Effective Rate, HAMD, Hamilton Anxiety Rating Scale (HAMA), Self-rating Anxiety Scale (SAS), SDS, Pittsburgh Sleep Quality Index (PSQI), Adverse effects and serum inflammatory factors: TNF-α and IL-6). Two assessors (JW and DZ.) independently evaluated study quality and risk of bias and extracted data using the Cochrane Collaboration Assessment Tool. Discrepancies between the assessors were resolved by a third assessor.

#### 2.1.4 Data analysis

STATA software (v16.0) to analyze this meta-analysis. For continuous outcomes (change in HAMD, SDS, HAMA, SAS, TNF-α, IL-6) and dichotomous outcomes (Total Effective Rate and Adverse Effects), the standardized mean difference (SMD) and risk ratios (RR) were represented, respectively. The 95% confidence interval (CI) was used to analyze the effects of secondary variables. When heterogeneity was high (*I* ^*2*^ > 50% or *p* < 0.05), a random effects model was used; otherwise, a fixed effects model was used. The subgroup analysis was based on the type of treatment group which included CLM + SSRI (CLM + SSRI vs. SSRI), CLM + SNRI (CLM + SNRI vs SNRI), and CLM (CLM vs. SSRI). Egger’s test and funnel plots were used to assess publication bias.

### 2.2 Network pharmacology

#### 2.2.1 Screening for compounds and corresponding targets of CLM

According to the 11 Chinese herbs included in CLM as recorded in the “Shang Han Lun”, the compounds and corresponding targets of each drug were screened from three databases TCMSP (https://tcmspw.com/tcmsp.php) (Zeng et al., 2022), the HERB database (http://herb.ac.cn/) (Fang et al., 2021), and the BATMAN-TCM database (http://bionet.ncpsb.org/batman-tcm/; score ≥20, *p*-value ≤0.05) (Gao et al., 2021). Subsequently, all screened ingredients were imported into the TCMSP for normalization according to ADME (absorption, distribution, metabolism, excretion) criteria. In addition, oral bioavailability ≥30% and drug-likeness ≥0.18 as the most commonly used pharmacokinetic parameters to measure drug properties.

#### 2.2.2 Target prediction for depression

The keyword “depression” was searched to obtain potential targets related to depression from the GeneCards (Stelzer et al., 2016) and DisGeNET (Pinero et al., 2017) databases. To forecast CLM’s therapeutic targets for treating depression, the common targets of compounds and depression were sorted out, and a Venn diagram was obtained using the Venn 2.1.0 platform (Heberle et al., 2015). The intersection targets were entered into Cytoscape software (v 3.8.2) to construct Herb-Compound-Target (H-C-T) networks.

#### 2.2.3 Gene ontology and kyoto encyclopedia of genes and genomes enrichment analyses

For KEGG and GO enrichment analyses, we uploaded intersection targets into the Metascape platform (http://metascape.org/ *Homo sapiens*, *p* < 0.05, and *p*-values were corrected by the Benjamini–Hochberg procedure) (Zhou et al., 2019).

#### 2.2.4 Construction of protein-protein interaction network and topological analysis

To further evaluate the core of regulatory targets in the treatment of depression and discover potential connections, intersection targets were uploaded to the STRING database (*H. sapiens*, minimum required interaction score: 0.9) to construct a PPI network (Szklarczyk et al., 2019). Subsequently, to evaluate the topological characteristics of the nodes, the three parameters of “betweenness centrality” (BC), “closeness centrality” (CC), and “degree centrality” (DC) were computed.

#### 2.2.5 Molecular docking verification

To assess the binding affinity of components with predicted targets, the active components of CLM were docked with core targets. The selection of active ingredients and core targets were as follows: 1) the degree values of H-C-T and PPI; 2) the correlation of enriched signal pathways according to GO and KEGG analysis. First, the core targets’ three-dimensional structure “PDB” files were retrieved from the PDB database (O'Boyle et al., 2011). Autodock Tools (v1.5.7) was used to remove the water molecules, original ligands, add hydrogens, calculate Gasteiger partial charges, and set atom types. The “SDF” format of the CLM bioactive component was obtained from the PubChem database (https://www.rcsb.org/), and the torsion angles in the ligand were identified, the solvent model was added, and the Kollman atomic charges were assigned to the protein using Autogrid4 and Autodock4. The “Local Search Parameters” algorithm was used for the docking operation. Finally, we constructed the docking interaction pattern diagram and displayed the docking findings via PyMoL (v2.4.0).

## 3 Results of meta-analysis

### 3.1 Study screening

**FIGURE 2:**
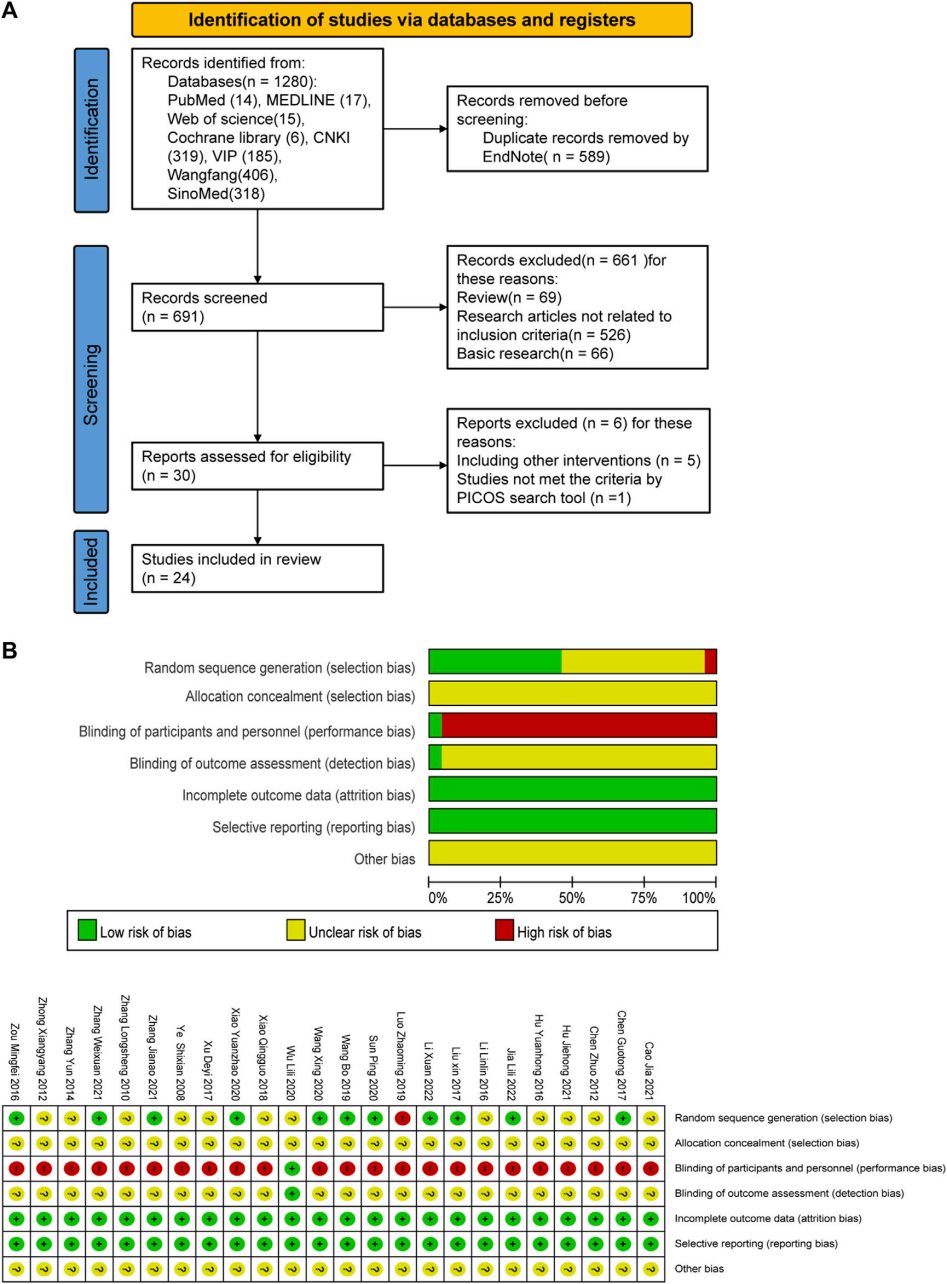
PRISMA flow diagram and risk-of-bias assessment; **(A)** literature screening process; **(B)** risk-of-bias summary.

A total of 1280 relevant original studies were retrieved from eight databases including PubMed (14 studies), MEDLINE (17), Web of Science (15), Cochrane Library (6), China National Knowledge Internet (319), VIP (185), Wan Fang (406), and Sino Med (318). There were 589 duplicates that were eliminated. Primary screening was then performed on the remaining 691 articles, and 661 trials were removed based on the abstracts and titles. Out of the remaining 30 articles, 24 items were included in this study. (Figure 2; Table 1).

**TABLE 1:** Basic characteristics of included studies

Study	Sample (T/C)	Male/female	Average age/years (T/C)	Interventions		Duration/weeks	Average course/years (T/C)	Outcomes measures
				T	C			
Jia and Li (2022)	51/51	55/47	40.12 ± 6.32/41.73 ± 6.79	CLM + SSRI	SSRI	6	1.53 ± 1.16/1.81 ± 1.57	①②⑦
Li X et al. (2022)	48/48	54/42	56.45 ± 4.56/57.01 ± 4.38	CLM	SSRI	8	6.32 ± 3.24/6.41 ± 2.32	①②⑤
Zhang et al. (2021)	20/20	23/17	35.48/36.24	CLM	SSRI	4	0.86/0.89	①②⑥⑦
Cao and Guo (2021)	61/61	57/56	-	CLM	SNRI	8	-	①②⑥
Hu (2021)	49/49	37/61	36.42 ± 6.93/37.82 ± 7.56	CLM + SSRI	SSRI	8	3.16 ± 1.09/3.62 ± 0.85	②
Zhang (2021)	55/55	23/32 20/35	46.59 ± 2.91/47.26 ± 2.74	CLM + SSRI	SSRI	4	-	④⑤⑦
Wang et al. (2020)	30/28	17/41	50.2/50.6	CLM	SSRI	2	3.11/3.46	①②⑥
Xiao and Han (2020)	64/64	63/65	43.15/43.46	CLM	SSRI	4	0.14/0.14	①④⑤⑦
Wu et al. (2020)	40/40	40/40	38.96/40.41	CLM	SSRI	4	6.03/5.81	①②⑤⑦
Sun (2020)	40/40	43/37	55.21 ± 2.74/55.41 ± 2.21	CLM + SNRI	SNRI	8	0.47 ± 0.03/0.47 ± 0.02	①②④⑤⑦
Wang et al. (2019)	100/100	84/116	39.5/39.5	CLM	SSRI	8	4.94/5.13	①②⑤
Luo et al. (2019)	42/38	36/44	39.5/39.6	CLM	SSRI	8	1.0/1.0	①②⑦
Xiao et al. (2018)	40 /38	34/44	35.24/37.56	CLM	SNRI	8	2.5/2.6	①②③
Xu and Zhao (2017)	40/40	20/60	42.5/41.1	CLM	SSRI	4	2.6/2.4	①⑧⑨
Liu et al. (2017)	34/34	31/37	42.1 ± 12.7/41.7 ± 13.1	CLM + SSRI	SSRI	6	-	①②⑧⑨⑦
Chen et al. (2017)	150/150	80/70 82/68	36.29 ± 3.72/37.07 ± 3.20	CLM + SSRI	SSRI	12	2.70 ± 1.01/2.82 ± 1.02	①④⑤
Li et al. (2016)	55/55	36/74	37.4/36.8	CLM	SSRI	8	3.4/3.2	②
Zou (2016)	42/41	46/37	37.34/37.02	CLM	SSRI	8	3.67/3.61	①②⑦
Hu et al. (2016)	30/30	-	-	CLM	SSRI	6	-	①②⑤⑦
Zhang (2014)	36/36	34/32	48.7	CLM	SSRI	8	4.65 ± 2.51	①②⑤
Chen and Ding (2012)	40/40	26/54	43.15 ± 11.74/44.25 ± 12.53	CLM	SSRI	8	0.5/0.5	①
Zhong et al. (2012)	50/50	42/58	51.34 ± 5.12/53.42 ± 4.31	CLM	SSRI	8	4.21 ± 2.16/3.86 ± 1.94	①②⑤
Zhang (2010)	31/32	24/39	36/39	CLM	SSRI	6	0.25/0.3	①②④⑤⑦
Ye and Luo (2008)	50/50	47/53	36.21 ± 16.03/34.78 ± 15.87	CLM	SSRI	4	0.5/0.5	①②③⑦

Note: T: treatment group; C: control group; CLM: Chaihu-jia-Longgu-Muli-tang; ①Clinical efficacy rate; ②HAMD Scores ; ③HAMA Scores; ④SAS Scores; ⑤SDS Scores; ⑥PSQI Scores; ⑦Adverse Effects; ⑧TNF-α; ⑨IL-6.

### 3.2 Characteristics and quality of study

Twenty-four RCTs with a total of 2,382 patients with depression were included (1195 cases in the treatment group, 1187 cases in the control group), involving five interventions, mainly including (SSRI, SNRI, CLM, CLM + SSRI, CLM + SNRI). According to the intervention in the treatment group, 13 trials on CLM + SSRI included 1337 patients, 3 trials on CLM + SNRI included 280 patients, and 8 trials on CLM alone included 765 patients. The treatment duration varies from 2 to 12 weeks.

A total of 21 trials discussed the total effective rate, of which 8 trials were for CLM alone, 10 trials were for CLM + SSRI, and 3 trials were for CLM + SNRI; 19 trials reported HAMD scores, among which 7 trials were for CLM alone, 10 trials were for CLM + SSRI, and 2 trials were for CLM + SNRI; 11 trials reported SDS scores, of which 6 trials were for CLM alone, 4 trials were for CLM + SSRI, and 1 trial was for CLM + SNRI; 2 trials reported HAMA scores, of which was for CLM and CLM + SNRI respectively; 5 trials reported SAS scores, among which 1 trial was for CLM alone, 3 trials were for CLM + SSRI, and 1 trial was for CLM + SNRI; 3 trials reported PSQI scores, all of included were for CLM + SSRI; 2 trials detected the serum levels of IL-6 and TNF-α for CLM + SSRI; and 12 trials assessed adverse effects after treatment, among which 2 trials were for CLM alone, 9 trials were for CLM + SSRI, and 1 trial were for CLM + SNRI. Additionally, Table 1 and Figure 2 show the basic characteristics of the studies and results of the risk-of-bias assessment.

### 3.3 Total effective rate

**FIGURE 3:**
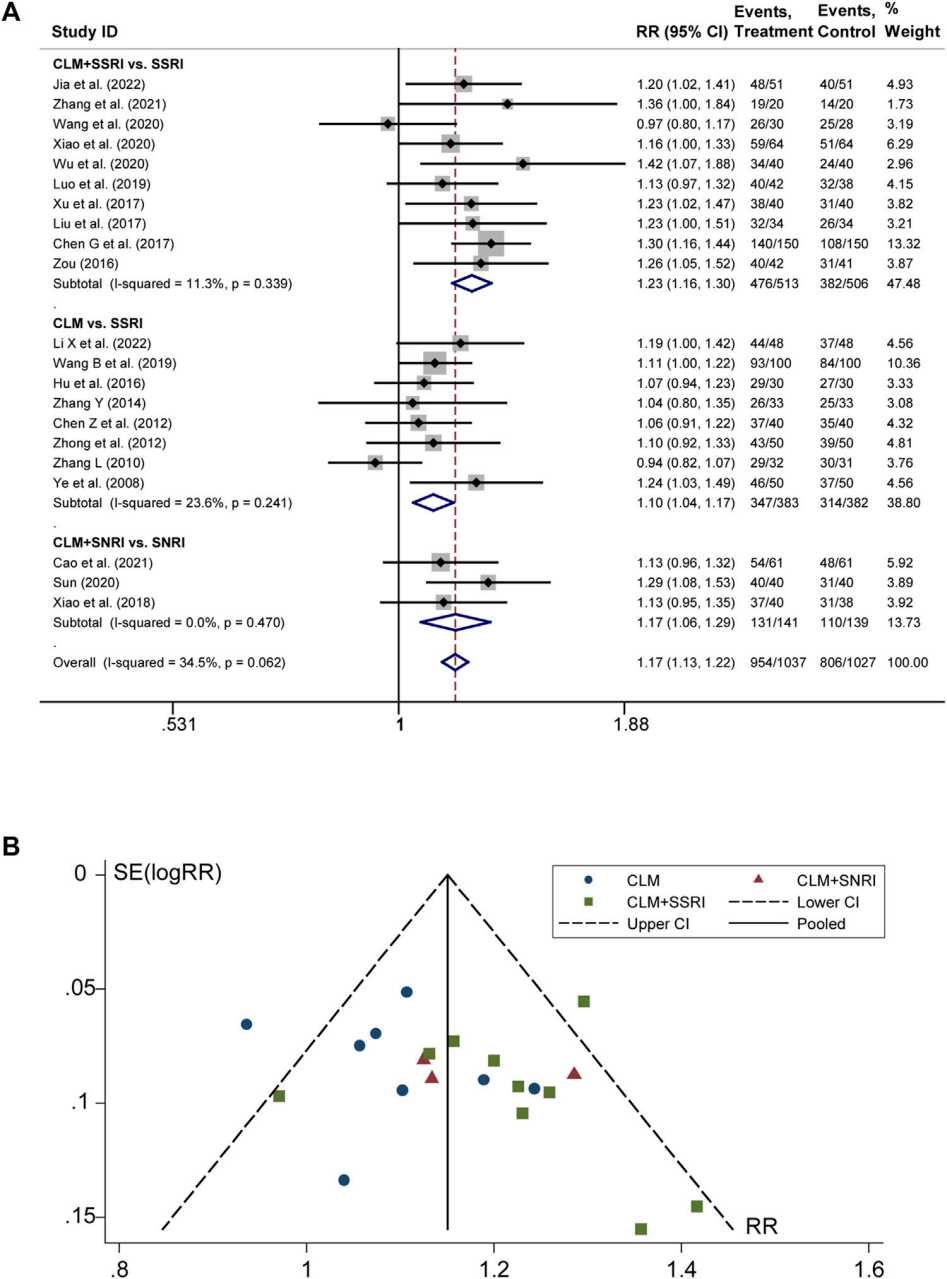
Comparative forest plots of total effective rate; **(A)** forest plot showing the total effective rate of CLM alone or anti-depressant drug therapy on depression; Treatment: CLM alone or combined with pharmaceutical anti-depressants; Control: pharmaceutical anti-depressants; **(B)** funnel plot of total effective rates.

21 studies discussed the effective rate of CLM for treating depression. According to heterogeneity testing (*p* = 0.062, *I* ^*2*^ = 34.5%), the fixed effects model was applied. The results indicated that the effective rate of the treatment group was significantly higher than that of the control group (RR = 1.17%, 95% CI [1.13, 1.22]; Figure 3A). For the subgroup analysis, the anti-depressive effect on CLM + SSRI, CLM, and CLM + SNRI is significantly better than that of SSRI or SNRI alone (RR = 1.23%, 95% CI [1.16, 1.30], *p* = 0.339, *I* ^*2*^ = 11.3%; RR = 1.10%, 95% CI [1.04, 1.17], *p* = 0.241, *I* ^*2*^ = 23.6%; RR = 1.17%, 95% CI [1.06, 1.29], *p* = 0.470, *I* ^*2*^ = 0.0%; Figure 3A). Moreover, the funnel plot symmetry indicated no publication bias (Egger’s test: *p* = 0.280, Figure 3B).

### 3.4 HAMD and self-rating depression scale scores

**FIGURE 4:**
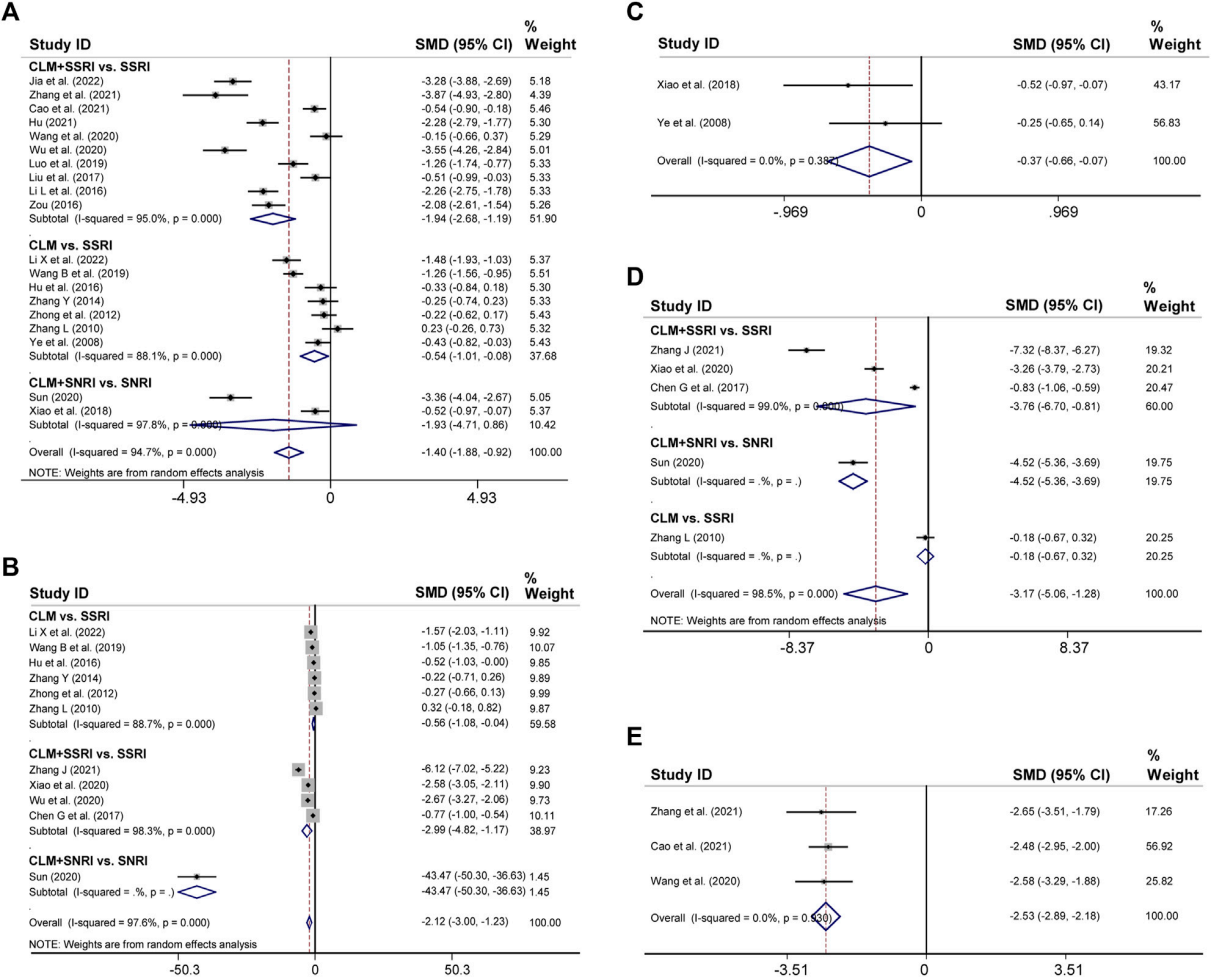
Comparative forest plots of HAMD, SDS, HAMA, SAS, and PSQI scores; **(A)** HAMD scores; **(B)** SDS scores; **(C)** HAMA scores; **(D)** SAS scores; **(E)** PSQI scores.

19 trials reported the post-treatment HAMD scores. According to heterogeneity testing (*p* = 0.000, *I* ^*2*^ = 94.7%), the random effects model was applied. Results indicated that the CLM treatment group showed more reduction in HAMD scores than the control group (SMD = −1.40%, 95% CI [−1.88, −0.92], Figure 4A). For the subgroup analysis, the CLM + SSRI, CLM, and CLM + SNRI showed significant reduction in HAMD scores comparing SSRI or SNRI alone (RR = −1.94%, 95% CI [−2.68, −1.19], *p* = 0.000, *I* ^*2*^ = 95.0%; RR = −0.54%, 95% CI [−0.101, −0.08], *p* = 0.000, *I* ^*2*^ = 88.1%; RR = −1.93%, 95% CI [−4.71, 0.86], *p* = 0.000, *I* ^*2*^ = 97.8%; Figure 4A).

Regarding SDS, 11 trials had high heterogeneity (*p* = 0.000, *I* ^*2*^ = 97.6%), and the random effects model was adopted. The results showed that the treatment group significantly improved negative emotions compared to the control group (SMD = −2.12%, 95% CI [−3, −1.23], Figure 4B). For the subgroup analysis, the CLM, CLM + SSRI, and CLM + SNRI showed significant reduction in SDS scores comparing SSRI or SNRI alone (RR = −0.56%, 95% CI [−1.08, −0.04], *p* = 0.000, *I* ^*2*^ = 88.7%; RR = −2.99%, 95% CI [−4.82, −1.17], *p* = 0.000, *I* ^*2*^ = 98.3%; RR = −2.12, 95% CI [−3.00, −1.23], *p* = Not applicable, *I* ^*2*^ = Not applicable; Figure 4B)

### 3.5 HAMA, self-rating anxiety scale, pittsburgh sleep quality index scores

Two trials reported HAMA scores for CLM and CLM + SNRI respectively. According to heterogeneity testing (*p* = 0.387, *I* ^*2*^ = 0.0%), the fixed effects model was applied. The HAMA scores of the group of patients receiving CLM or combined with antidepressant drugs were significantly lower than those of the control group (SMD = −0.37%, 95% CI [−0.66, −0.07], Figure 4C).

11 trials reported SAS scores. According to heterogeneity testing (*p* = 0.000, *I* ^*2*^ = 98.5%), the random effects model was performed. The SAS scores of the treatment group were significantly lower than those of the control group (SMD = −3.17%, 95% CI [−5.06, −1.28], Figure 4D). For the subgroup analysis, the CLM + SSRI, CLM + SNRI, and CLM showed marked reduction in SAS scores comparing SSRI or SNRI alone (RR = −3.76%, 95% CI [−6.70, −0.81], *p* = 0.000, *I* ^*2*^ = 99.0%; RR = −4.52%, 95% CI [−5.36, −3.69], *p* = Not applicable, *I* ^*2*^ = Not applicable; RR = −0.18%, 95% CI [−0.67, −0.32], *p* = Not applicable, *I* ^*2*^ = Not applicable; Figure 4B)

Three trials reported PSQI scores, all of included were for CLM + SSRI. According to heterogeneity testing (*p* = 0.930, *I* ^*2*^ = 0.0%), the fixed effects model was applied. The PSQI scores of the treatment group were significantly lower than those of the control group (SMD = −2.53, 95% CI [−2.89, −2.18], Figure 4E).

### 3.6 Inflammation index

**FIGURE 5:**
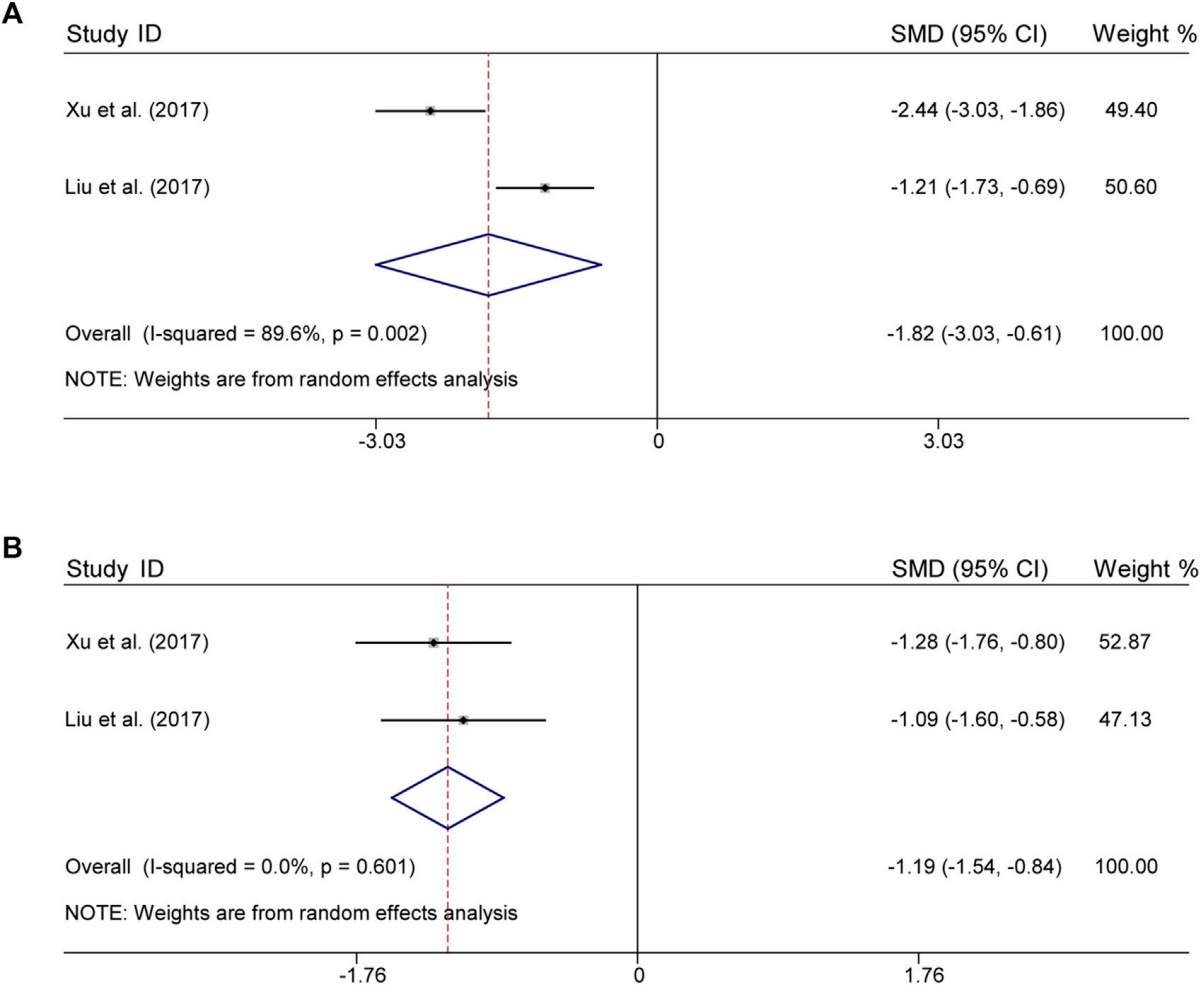
Comparative forest plots of indicators of inflammation; **(A)** TNF-α; **(B)** IL-6.

There were 2 studies both for CLM + SSRI that assessed serum levels of TNF-α and IL-6. For TNF-α, according to heterogeneity testing (*p* = 0.002, *I* ^*2*^ = 89.6%), the random effects model was applied, which in the treatment group were significantly lower than those in the control group (SMD = −1.82%, 95% CI [−3.03, −0.61], Figure 5A). For IL-6, according to heterogeneity testing (*p* = 0.601, *I* ^*2*^ = 0.0%), the fixed effects model was applied. Compared with the control group, the treatment group showed significantly lower levels (SMD = −1.19%, 95% CI [−1.54, −0.84], Figure 5B).

### 3.7 Adverse effects

**FIGURE 6:**
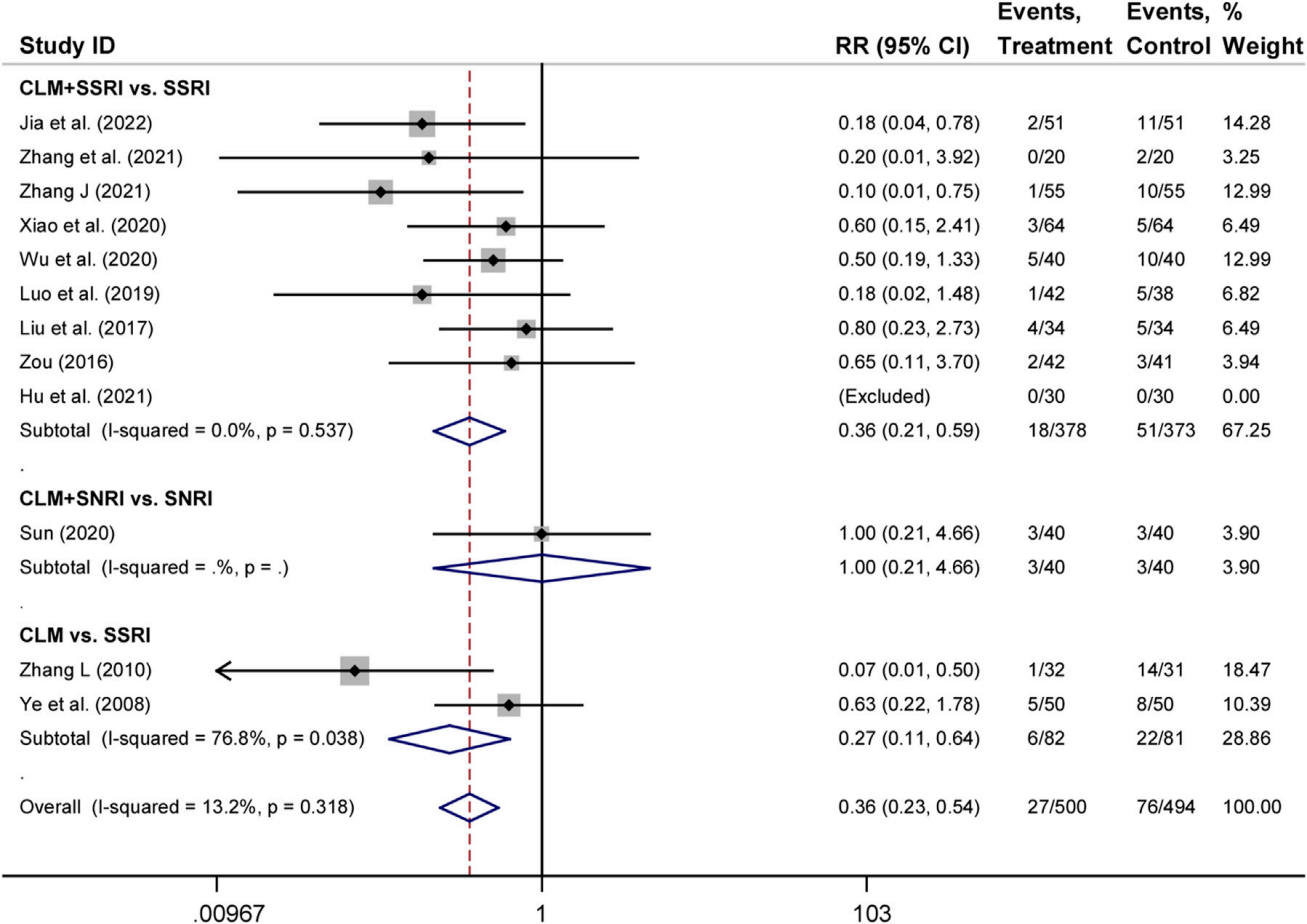
Comparative forest plots of adverse effects; Treament: CLM alone or combined with pharmaceutical anti-depressants; Control: pharmaceutical anti-depressants.

There were 12 trials that assessed adverse occurrences in the 24 included studies, covering neurological symptoms such as dizziness, headache, sleep disorders, hyperactivity, blurred vision, and fatigue, as well as gastrointestinal symptoms such as dry mouth, diarrhea, anorexia, nausea, vomiting, and constipation. The most common adverse reactions were dizziness, blurred vision, dry mouth, anorexia, and vomiting. According to heterogeneity testing (*p* = 0.250, *I* ^*2*^ = 13.2%), the fixed effects model was applied. The treatment group showed better safety in treating depression than the control group (RR = 0.36%, 95% CI [0.23, 0.54], Figure 6). For the subgroup analysis, CLM + SSRI and CLM alone showed fewer adverse effects compared the SSRI alone (RR = 0.36%, 95% CI [0.21, 0.59], Figure 6), however, there was no statistical significance between CLM + SNRI and SNRI alone.

## 4 Results of network pharmacology analysis

### 4.1 Active compounds and treatment targets of CLM

We obtained 129 active compounds from the four databases, including Radix Bupleuri (16 compounds), Radix Scutellariae (29 compounds), Pinelliae Tuber (10 compounds), Ginseng radix (35 compounds), Hoelen (12 compounds), Zingiber rhizoma (5 compounds), Zizyphi fructus (25 compounds), Radix et Rhizoma Rhei (7 compounds), and Cinnamomi cortex (7 compounds). The primary active components of CLM are listed in Supplementary Table S3.

**FIGURE 7:**
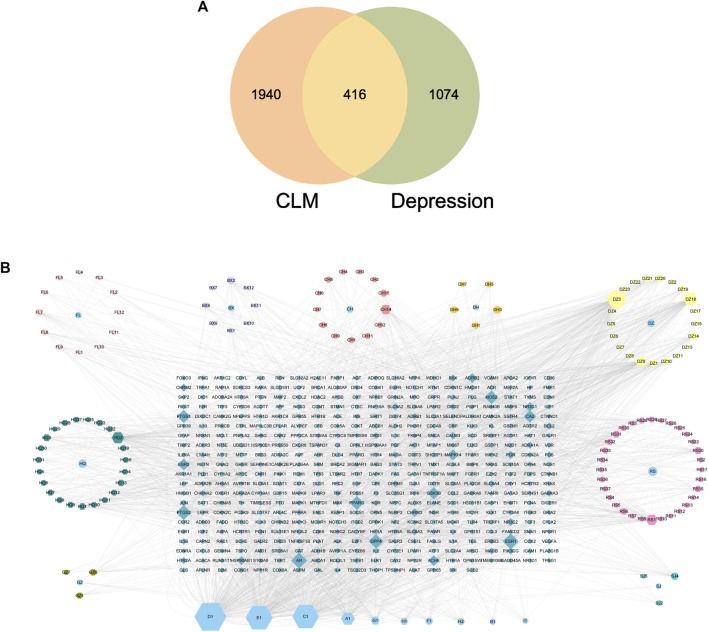
Venn diagram and H-C-T network; **(A)** Venn diagram of the active ingredients of CLM and potential treatment targets; 2356 predicted targets of CLM, 1490 therapeutic targets for depression, and 416 targets intersected; **(B)** H-C-T network of CLM; BX: Ban Xia, CH: Chai Hu, DZ: Da Zao, FL: Fu Ling, SJ: Sheng Jiang, GZ: Gui Zhi, DH: Da Huang, HQ: Sheng Jiang, RS: Ren Shen; A–H: Common components of different drugs.

There were 2356 targets screened from the 129 active compounds CLM using the Swiss, TCMSP, HERB, and BATMAN-TCM databases. In addition, 1490 depression-related targets were identified using the DisGeNET and GeneCards databases. The intersection of drug targets and depression targets resulted in a total of 416 CLM treatment depression targets, as shown in the Venn diagram (Figure 7A).

### 4.2 The construction of the H-C-T network

The H-C-T network included 555 nodes and 2480 edges and contained nine herbs, 130 compounds, and 416 genes (Figure 7B). Larger nodes have higher statistical significance. The top five compounds, as determined by degree analysis, were MOL000449 (stigmasterol), MOL000098 (quercetin), MOL000358 (β-stigmasterol), MOL012976 (coumestrol), and MOL000627 (stepholidine), with respective degrees of 245°, 199°, 196°, 130°, and 85°. More details are given in Table 2.

**TABLE 2:** Basic information of the top five degree of the compounds.

MOL ID	Compound	CAS	Molecular formula	MW
MOL000449	Stigmasterol	83–48–7	C_29_H_48_O	412.77
MOL000098	Quercetin	117–39–5	C_15_H_10_O_7_	302.23
MOL000358	beta-stiosterol	83–46–5	C_29_H_50_O	414.79
MOL012976	coumestrol	479–13–0	C_15_H_8_O_5_	268.22
MOL000627	Stepholidine	16,562–13–3	C_19_H_21_NO_4_	327.41

### 4.3 Gene ontology and kyoto encyclopedia of genes and genomes enrichment analyze

**FIGURE 8:**
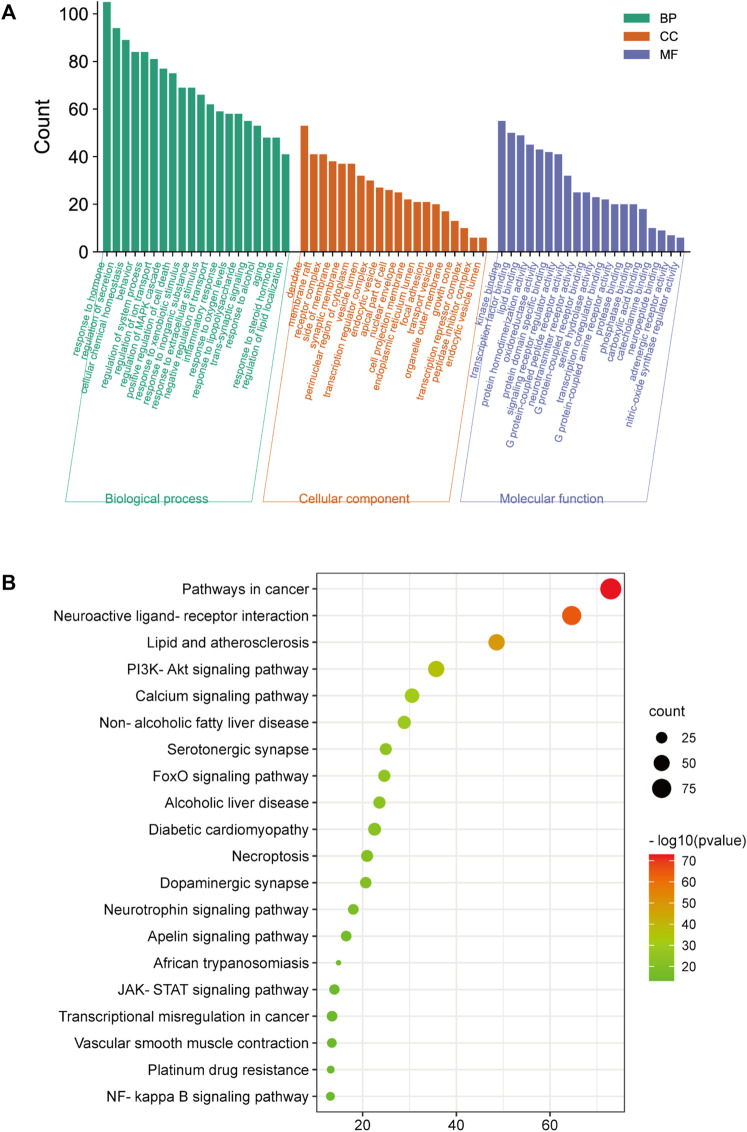
GO and KEGG analysis; **(A)** BP, MF, and CC categories in GO analysis of 416 intersection targets; **(B)** top 20 signaling pathways in KEGG enrichment analysis of 416 intersected targets.

For GO enrichment analysis, a total of 3516 items were selected, and the top 20 significant terms in the BP, CC, and MF categories are shown in Figure 8A. The intersected target proteins in the BP category mainly included responses to hormones, behavior, positive regulation of cell death, inflammatory response, and synaptic signaling. The CC category mainly included dendrite, cell body, membrane raft, perinuclear region of cytoplasm, and side of membrane. The MF category mainly included kinase binding, transcription factor binding, protein domain-specific binding, signaling receptor regulator activity, and neurotransmitter receptor activity.

For KEGG enrichment analysis, the top 20 items were selected for visualization according to the criteria above (Figure 8B). The results mainly involved neuroactive ligand-receptor interactions, the PI3K-Akt signaling pathway, serotonergic synapses, the JAK-STAT signaling pathway, and the NF-κB signaling pathway.

### 4.4 Protein-protein interaction network analysis

**FIGURE 9:**
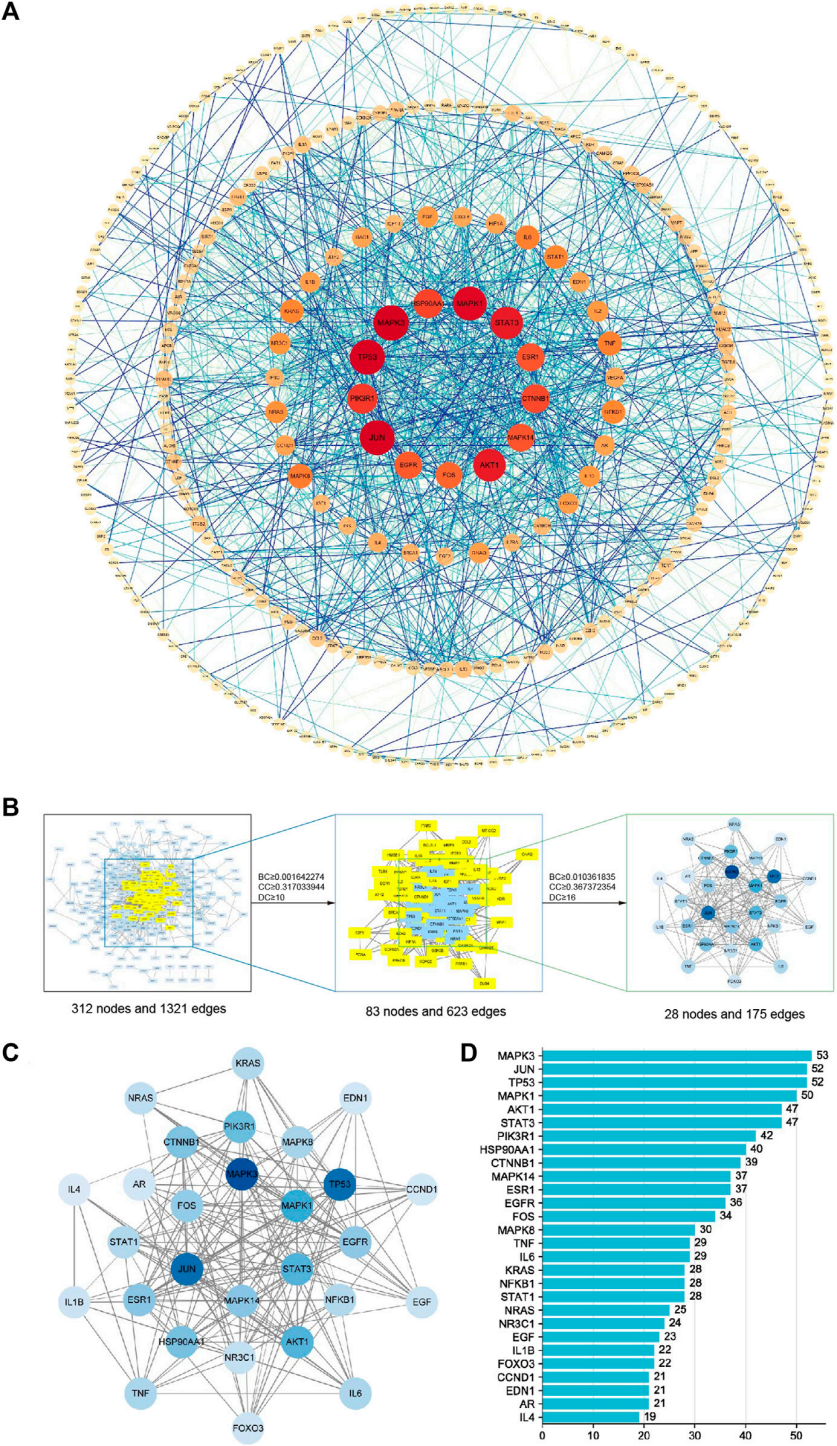
Interaction network diagram of 416 predicted targets and topology analysis; **(A)** PPI network of 416 predicted targets; **(B)** topology analysis; BC: betweenness centrality; CC: closeness centrality; DC: degree centrality; **(C)** network of the top 28 core proteins; **(D)** top 28 targets ranked by degree values.

In the PPI network analysis, 416 predicted targets were submitted to the STRING platform, and interactions with high confidence (>0.9) were chosen. Isolated nodes were removed, and the remaining targets were visualized in Cytoscape 3.8.2. The PPI relationship network, which consisted of 312 nodes and 1321 edges, is shown in Figure 9A. In addition, to further screen for key targets for CLM in the treatment of depression, we conducted topology analysis based on BC, CC, and DC. Ultimately, 28 core targets were obtained and ranked by degree values (Figures 9B–D; Table 3). The top seven core targets were MAPK3, JUN, TP53, MAPK1, AKT1, STAT3, and PIK3R1.

**TABLE 3:** Specifc information of the 28 key target genes.

Uniprot ID	Name	Description	Degree	Betweenness centrality	Closeness centrality
P27361	MAPK3	Mitogen-activated protein kinase 3	53	0.045746269	0.435103245
P05412	JUN	Transcription factor Jun	52	0.084722588	0.44494721
P04637	TP53	Cellular tumor antigen p53	52	0.120920491	0.435745938
P28482	MAPK1	Mitogen-activated protein kinase 1	50	0.038943892	0.431918009
P40763	STAT3	Signal transducer and activator of transcription 3	47	0.058421284	0.421428571
P31749	AKT1	RAC-alpha serine/threonine-protein kinase	47	0.071162417	0.43318649
P27986	PIK3R1	Phosphatidylinositol 3-kinase regulatory subunit alpha	42	0.046009982	0.416666667
P07900	HSP90AA1	Heat shock protein HSP 90-alpha	40	0.0405667	0.411436541
P35222	CTNNB1	Catenin beta-1	39	0.059706312	0.411436541
Q16539	MAPK14	Mitogen-activated protein kinase 14	37	0.069980098	0.431918009
P03372	ESR1	Estrogen receptor	37	0.081274348	0.43255132
P00533	EGFR	Epidermal growth factor receptor	36	0.033559803	0.412011173
P01100	FOS	Protein c-Fos	34	0.024626294	0.408587258
P45983	MAPK8	Mitogen-activated protein kinase 8	30	0.032364484	0.409722222
P05231	IL6	Interleukin-6	29	0.01798382	0.380645161
P01375	TNF	Tumor necrosis factor	29	0.027560775	0.387139108
P19838	NFKB1	Nuclear factor NF-kappa-B p105 subunit	28	0.032042801	0.402455662
P42224	STAT1	Signal transducer and activator of transcription 1-alpha/beta	28	0.015860428	0.398648649
P01116	KRAS	GTPase KRas	28	0.017076325	0.39021164
P01111	NRAS	GTPase NRas	25	0.024069906	0.37966538
P04150	NR3C1	Glucocorticoid receptor	24	0.014751511	0.399188092
P01133	EGF	Pro-epidermal growth factor	23	0.014372036	0.380154639
O43524	FOXO3	Forkhead box protein O3	22	0.02241392	0.397039031
P01584	IL1B	Interleukin-1 beta	22	0.012354862	0.380645161
P24385	CCND1	G1/S-specific cyclin-D1	21	0.014984121	0.383615085
P10275	AR	Androgen receptor	21	0.016074494	0.392287234
P05305	EDN1	Endothelin-1	21	0.020720848	0.386125654
P05112	IL4	Interleukin-4	19	0.037711426	0.381630013

### 4.5 Molecular docking

**FIGURE 10:**
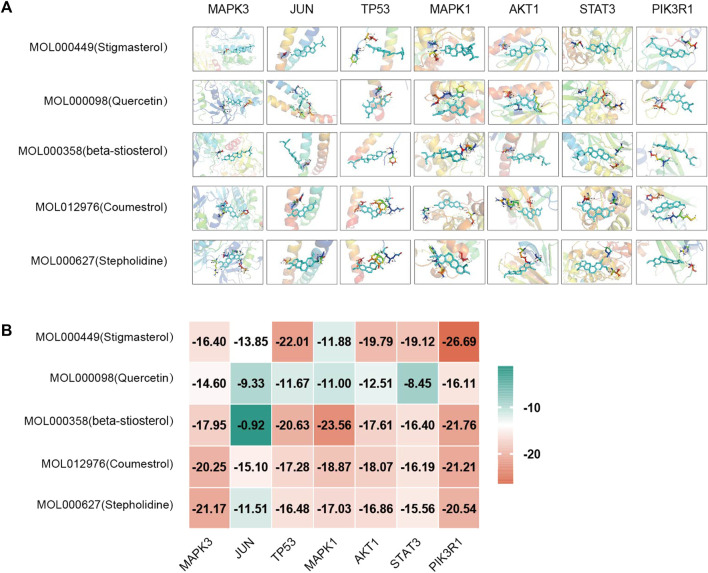
Molecular docking analysis of main compounds binding to key targets; **(A)** molecular docking models of main chemical components; **(B)** heat map showing values of docking affinity.

Based on the above results in H-C-T, GO, and PPI network analysis, molecular docking analysis was performed between the top five compounds of CLM and the top seven key targets. The docking visualizations and scores are shown in Figures 10A,B. The stronger the binding force between the compound and the protein, the lower the docking score (the higher the negative number). When the docking score is ≤ −5.0 kJ/mol, the exertion of a strong binding effect is suggested. The results showed that 35 pairs of active compounds and ligands were relatively stable. PIK3R1 (PDB: 2IUG), MAPK3 (PDB: 4QTB), and AKT1 (PDB: 1UNP) might be potential therapeutic targets for depression using CLM, among which stigmasterol (MOL000449) had the highest binding energy with PIK3R1.

## 5 Discussion

At present, the pathological mechanisms of depression mainly involve the monoamine hypothesis, hypothalamus–pituitary–adrenal axis hyperactivity, neuroplasticity and neurogenesis, structural and functional brain changes, and inflammation (Malhi and Mann, 2018). However, due to the unclear pathophysiological mechanisms of depression, one-third of patients face treatment resistance or adverse reactions to common treatments, and the treatment of depression still faces challenges (Dodd et al., 2021). CLM is derived from “Shang Han Lun” and has a significant effect on depression. However, due to the complexity of its components, the mechanism of action of this prescription has not been fully clarified. The results of present study indicated that the antidepressant efficacy of CLM, whether administered alone or in combination with antidepressants, was significantly higher than that of antidepressants alone. In addition, whether in CLM alone or CLM + SSRI/SNRI, the symptoms of depression, anxiety, and insomnia in patients with depression were significantly improved comparing antidepressants alone. CLM and CLM + SSRI also showed fewer adverse reactions than SSRI alone. Five compounds in CLM and seven of its key targets were shown to be related to antidepressant activity in the present analysis.

CLM has extensive neural activities in the treatment of depression, anxiety, insomnia, and neurocognitive disorders (Niitsu et al., 2013). A meta-analysis of CLM’s efficiency and safety in treating post-stroke depression revealed that, regardless of the length of the treatment period, CLM showed better efficacy than an antidepressant group. Additionally, in the group receiving CLM combined with pharmaceutical antidepressants, the incidence of adverse reactions such as abnormal blood or urine routine tests, abnormal liver function, sleeplessness, and digestive tract pain was dramatically decreased (Wan et al., 2021). In addition, a correlation has been shown between depression, anxiety, and insomnia; the association between insomnia and feelings of self-disgust was fully mediated by anxiety and depression (Ypsilanti et al., 2018). This means that a good treatment strategy for patients with depression seems to be relieving anxiety and insomnia while adjusting mood. In the present findings, compared with antidepressants, both CLM alone and combined with SSRI/SNRI markedly reduced the scores of HAMD and SDS. For anxiety and insomnia, we conducted a meta-analysis of HAMA, SAS, and PSQI scores, results indicated that the synergistic effect of CLM and SSRI was significantly better than that of SSRI alone. In addition, due to the limited literature on CLM alone and CLM + SNRI, the synergistic effect of CLM alone or with SNRI may need further exploration. However, for overall efficacy in anxiety, CLM alone or in combination is not inferior to antidepressants alone. In terms of adverse reactions, whether CLM alone or in combination with SSRI, the incidence of adverse reactions is significantly fewer than that of SSRI alone. It is worth noting that the incidence of adverse reactions in CLM combined with SNRI is the same as that of SNRI alone, which may be the reason for only including one trial. Therefore, CLM could not only improve the symptoms of depression, anxiety, and insomnia, but can also reduce the incidence of adverse responses caused by commonly used antidepressants, especially in combination with SSRI.

Depression and inflammation are intertwined; heightened inflammation exacerbates not only physical symptoms such as pain sensitivity, fatigue, and anhedonia but also psychological symptoms such as negative mood, loss of appetite, and feelings of inferiority (Kiecolt-Glaser et al., 2015). Levels of proinflammatory cytokines in peripheral blood were shown to be significantly increased in patients with major depressive disorder, such as IL-6, TNF-α and IL-1β (Howren et al., 2009; Dowlati et al., 2010; Liu et al., 2012). Moreover, nonsteroidal anti-inflammatory drugs could significantly reduce depressive symptoms compared to placebo treatment (Kohler et al., 2014). Therefore, anti-inflammatory treatments may be effective for depression. The present results indicated that CLM dramatically decreased the levels of TNF-α and IL-6 in the peripheral blood of patients with depression compared to antidepressant treatment groups.

In order to further investigate the aforementioned putative mechanisms of CLM’s anti-depressant effects, network pharmacology was conducted. We intersected 416 therapeutic targets from 1490 depression targets and 2356 targets of CLM compound components. According to the H-C-T network, the top five active ingredients were stigmasterol, quercetin, β-stiosterol, coumestrol, and stepholidine. Recent research has shown that oral and intraperitoneal treatment of stigmasterol and β-stiosterol significantly reduced immobility time in tail suspension and forced swimming tests in rat models of depression, and its antidepressant activity might be mediated by the glutamatergic systems (Zhao et al., 2016; Ghosh et al., 2022). Quercetin has been reported to inhibit inflammation and attenuate depression-like behavior by regulating PI3K/AKT/NF-κB and promoting mitophagy in a lipopolysaccharide-induced mouse model of depression (Han et al., 2021; Sun et al., 2021). Monoamine oxidase A inhibitors, as third-line antidepressants, are widely used in clinics, and coumestrol has been proven to be a selective and competitive monoamine oxidase A inhibitor (Seong et al., 2022). As a specific dopamine receptor D1 agonist, stepholidine could activate the PKA/mTOR pathway to upregulate the expression of synaptogenesis-related proteins and exert antidepressant effects (Zhang et al., 2017). The above research provides a pharmacological basis for the clinical efficacy of CLM in depression, although the effect of CLM on depression has been revealed directly or indirectly through the above ingredients, due to the complexity of the ingredients, the specific mechanisms have not been clarified. Therefore, we assessed the relevant pathways for CLM in the treatment of depression using KEGG and GO enrichment analyses.

Research has confirmed that inflammation induced by glial cells is an important mechanism leading to depression. Neuroglial cells can regulate neuron electrical activity and even death by adjusting synaptic plasticity and integrating and transmitting synaptic information (Perez-Catalan et al., 2021). This may explain the anti-inflammatory and antidepressant mechanism of CLM, where GO analysis showed that the antidepressant effect of CLM mainly plays a protective role by regulating the inflammatory response. In addition, at the level of CC and MF, CLM mainly plays a role in synaptic spines, synapses, membrane rafts, and nuclear envelope by combining with enzymes and neurotransmitters. The present KEGG analysis of CLM also pointed to neuroinflammation, which involves the PI3K/Akt, JAK-STAT, and NF-κB signaling pathways. The PI3K/Akt signaling pathway is involved in synaptic plasticity, learning and memory, and inflammation, which are important in the pathogenesis of depression (Matsuda et al., 2019). Enzymatic activity of PI3K and Akt was shown to be decreased in patients with depression, which may lead to neuron loss, decreased neuroplasticity, and dysregulation of neurotrophic factors (Karege et al., 2011). Evidence has also shown that activation of Akt signaling can alleviate stress-induced depressive behaviors in mice by inhibiting neuroinflammation and increasing neurotrophic factors (Xian et al., 2019). Accordingly, by activating Akt signaling, CLM was shown to reverse the abnormal expression of AMPA and NMDA receptors in the prefrontal cortex of depressed mice (Wang et al., 2018). The JAK-STAT and NF-κB signaling pathways are downstream cascade signals of the PI3K/Akt pathway and both play key roles in cell proliferation, differentiation, migration, and apoptosis. The involvement of the JAK/STAT pathway in synaptic plasticity has been confirmed to depend on the activation of NMDA receptors in the hippocampal CA1 region (Nicolas et al., 2012). Therefore, we speculate that the antidepressant effect of CLM may be related to the activation of PI3K/Akt and its downstream signaling pathways, inhibition of inflammatory storms, and protection of neurons.

Notably, the therapeutic efficacy of ketamine, an NMDA receptor antagonist, has highlighted the emerging data linking the regulation of NMDA to the etiology of depression. More significantly, increases in proinflammatory cytokines (such as IL-1, IL-6, and TNF-α) have been directly associated with JAK-STAT activation. Antidepressants may work by decreasing proinflammatory cytokines via modulation of the JAK/STAT pathway, and they may also improve somatic symptoms, anhedonia, and low energy levels (Malemud and Miller, 2008). The NF-κB signaling pathway contributes to the pathogenesis of neurodegenerative diseases, with the PI3K/Akt pathway as its major upstream component (Dong et al., 2016). Activation of the PI3K/Akt signaling pathway has been reported to significantly reduce the nuclear translocation of NF-κB and the activation of microglia, thus inhibiting neuronal apoptosis and inflammation (Zhu et al., 2018). Although CLM likely exerts anti-depressant effects via the PI3K/Akt, JAK/STAT, NF-κB, or apoptosis signaling pathways, it remains urgently necessary to clarify the therapeutic targets of CLM, which may contribute to the development of novel therapeutic strategies.

Next, according to the PPI network and molecular docking analyses, MAPK3, TP53, MAPK1, STAT3, and PI3KR1 were potential antidepressant targets of CLM. Similar to the above results from the network pharmacology analysis, stigmasterol showed high binding energy with PI3KR1, suggesting that CLM alleviates depression via modulation of the PI3K/Akt signaling pathway. In summary, the above evidence suggests that CLM is a safe and effective antidepressant prescription with multiple pathways and targets. Its mechanism is likely related to the regulation of neuronal synaptic plasticity, inflammation, and apoptosis.

## 6 Limitations

This study still exist limitations. The treatment time was not explored on anti-depression efficacy. Secondly, it is still shallow that the evidence for selecting serum IL-6 and TNF-α as inflammation indicators. On the one hand, it is limited by available studies, on the other hand, other indicators such as serum IL-1β and CRP also affect the resistance to antidepressants. Meanwhile, the increase in these indicators also indicates more severe disease course outcomes and more extensive somatization symptoms (Kiecolt-Glaser et al., 2015). Furthermore, the number of trials included in this study was insufficient to clearly state the specific efficacy of some subgroup analyses such as HAMA and SAS, so part results have to be roughly concluded by assessing the comprehensive efficacy of CLM alone or in combination with antidepressants. Then, part of literature included by allocation concealment and blinding is not yet clear. TCM has the characteristics of personalized treatment, which is limited to the unique odor and color, making it difficult to implement blind methods. In addition, screening the active ingredients based on OB and DL may exist inconsistency with the precise ingredients, and the predicted targets are likely to be limited by the hot spots of current research. Finally, the specific mechanism in this study still needs to be verified by further vivo and vitro experiments.

## 7 Conclusion

This study analyzed the effectiveness and mechanism of CLM in the treatment of depression. Our findings revealed that compared to antidepressants, CLM had better clinical efficacy and fewer side effects, especially in combination with SSRI. Its mechanism in treating depression may be related to regulating neuronal synaptic plasticity, inhibiting neuroinflammation, and improving neuronal damage. Stigmasterol, β-stiosterol, and coumestrol, three components of CLM. may play antidepressant roles via the targets of PIK3R1, MAPK3, and AKT1. The PI3K/Akt signaling pathway may be one of the main pathways for CLM to achieve its antidepressant effect.

## Data Availability

The original contributions presented in the study are included in the article/Supplementary Materials, further inquiries can be directed to the corresponding authors.
