# Supplementary material for: The pharmacological mechanism of chaihu-jia-longgu-muli-tang for treating depression: integrated meta-analysis and network pharmacology analysis
Source: Front Pharmacol. 2023 Sep 21;14:1257617. doi: 10.3389/fphar.2023.1257617 (PMC10551636; doi:10.3389/fphar.2023.1257617)
Supplement: Supplementary file 1 [file DataSheet1.docx]

Supplementary Material

The pharmacological mechanism of Chaihu-jia-Longgu-Muli-tang for treating depression: Integrated meta-analysis and network pharmacology analysis

Yang Zhao^1†^, Dan Xu^2,3†^, Jing Wang^4^, Dandan Zhou^1^, Anlan Liu^1^, Yingying Sun^1^, Yuan Yuan^1^, Jianxiang Li^5*^, Weifeng Guo^1*^

*** Correspondence:**

Weifeng Guo

gwfwfg2003@njucm.edu.cn

Jianxiang Li

ljx029504@njucm.edu.cn

**^†^** These authors contributed equally to this work and shared the first authorship

**Supplementary Table 1**. The search strategy

| **Database** | **Search term** | **Search record** |
| --- | --- | --- |
| **PubMed/Medline** | ((Depression[Title/Abstract]) OR (Depressive[Title/Abstract])) AND ((((CLM[Title/Abstract]) OR (chaihujialonggumuli[Title/Abstract] OR chaihu jia longgu tang[Title/Abstract])) OR (chaihu jia longgu muli decoction[Title/Abstract])) OR (chaihu jia longgumuli formula[Title/Abstract])) | 14/17 |
| **Cochrane** | #1  (CLM):t,ab,kw OR (chaihujialonggumuli):ti,ab,kw OR (chaihu jia longu muli tang):ti,ab, kw OR (chaihu jia longu muli decoction):ti,ab,kw OR (chaihu jia longu muli formula):ti,ab,kw  #2  (depression):ti,ab,kw OR (depressive):ti,ab,kw  #3  #1 AND #2 | 6 |
| **Web of science** | #1  Depression (Topic) or Depressive (Topic)  #2  CLM (Topic) or chaihujialonggumuli (Topic) or chaihu jia longgu tang (Topic) or chaihu jia longgu muli decoction (Topic) or chaihu jia longgumuli formula (Topic)  #3  #1 AND #2 | 15 |
| **CNKI** | (主题 = 柴胡加龙骨牡蛎汤 OR 主题 = 柴胡龙牡汤 OR 主题 = 柴胡加龙牡汤 OR 主题 = 柴胡桂枝龙牡汤) AND (主题 = 抑郁 OR 主题 = 抑郁症 OR 主题 = 抑郁状态) | 319 |
| **VIP** | (M = 柴胡加龙骨牡蛎汤 OR M = 柴胡龙牡汤 OR M = 柴胡加龙牡汤 OR M = 柴胡桂枝龙牡汤) AND (M = 抑郁 OR M = 抑郁症 OR M = 抑郁状态) | 185 |
| **WanFang** | 主题:("柴胡加龙骨牡蛎汤" OR "柴胡龙牡汤" OR "柴胡加龙牡汤" OR "柴胡桂枝龙牡汤") AND 主题:("抑郁" OR "抑郁症" OR "抑郁状态") | 406 |
| **SinoMed** | (主题 = 柴胡加龙骨牡蛎汤 OR 主题 = 柴胡龙牡汤 OR 主题 = 柴胡加龙牡汤 OR 主题 = 柴胡桂枝龙牡汤) AND (主题 = 抑郁 OR 主题 = 抑郁症) | 318 |

**Table 2.** The composition of CLM.

| Local name | English name | Latin name | Part used | Origin (P. R.  China) |
| --- | --- | --- | --- | --- |
| Chai Hu | Bupleuri radix | Bupleurum chinense DC. | Root | Hu Bei |
| Huang Qin | Scutellariae Radix | Scutellaria baicalensis Georgi | Root | He Bei |
| Ban Xia | Pinelliae tuber | Pinellia ternata (Thunb.) Makino | Root | He Bei |
| Ren Shen | Ginseng radix | Panax ginseng C.A.Mey. | Root | Liao Ning |
| Sheng Jiang | Zingiberis rhizoma | Zingiber Officinale Roscoe | Root | He Bei |
| Da Zao | Zizyphi fructus | Ziziphus jujuba Mill. | Seed | Xin Jiang |
| Da Huang | Radix et Rhizoma Rhei | Rheum palmatum L. | Root | Shan Xi |
| Gui Zhi | Cinnamomi cortex | Cinnamomum cassia (L.) J.Presl | Root | Guang Dong |
| Fu Ling | Hoelen | Poria Cocos(Schw.) Wolf. | Fungi | Yun Nan |
| Sheng Longgu | Fossilia ossis mastoid |  | Bone | He Bei |
| Sheng Muli | Ostreae testa |  | shellfish | He Bei |

**Table 3.** 129 main active ingredients of CLM

| **Durg** | **Mol ID** | **Molecule Name** | **Durg** | **Mol ID** | **Molecule Name** |
| --- | --- | --- | --- | --- | --- |
| BX1 | MOL001755 | 24-Ethylcholest-4-en-3-one | GZ1 | MOL001736 | (-)-taxifolin |
| BX2 | MOL002670 | Cavidine | GZ1 | MOL001736 | (-)-taxifolin |
| BX7 | MOL005030 | gondoic acid | GZ6 | MOL004576 | taxifolin |
| BX8 | MOL000519 | coniferin | GZ7 | MOL011169 | Peroxyergosterol |
| BX9 | MOL006936 | 10,13-eicosadienoic | DH1 | MOL002235 | EUPATIN |
| BX10 | MOL006937 | 12,13-epoxy-9-hydroxynonadeca-7,10-dienoic acid | DH3 | MOL002268 | rhein |
| BX11 | MOL003578 | Cycloartenol | DH5 | MOL002281 | Toralactone |
| BX12 | MOL006967 | beta-D-Ribofuranoside, xanthine-9 | DH7 | MOL002297 | Daucosterol_qt |
| CH1 | MOL013187 | Cubebin | DH9 | MOL000471 | aloe-emodin |
| CH2 | MOL004718 | α-spinasterol | HQ1 | MOL001689 | acacetin |
| CH3 | MOL004702 | saikosaponin c_qt | HQ2 | MOL000173 | wogonin |
| CH4 | MOL004653 | (+)-Anomalin | HQ3 | MOL000228 | (2R)-7-hydroxy-5-methoxy-2-phenylchroman-4-one |
| CH5 | MOL004644 | Sainfuran | HQ5 | MOL002909 | 5,7,2,5-tetrahydroxy-8,6-dimethoxyflavone |
| CH6 | MOL004628 | Octalupine | HQ6 | MOL002910 | Carthamidin |
| CH7 | MOL004624 | Longikaurin A | HQ7 | MOL002913 | Dihydrobaicalin_qt |
| CH8 | MOL004609 | Areapillin | HQ8 | MOL002914 | Eriodyctiol (flavanone) |
| CH9 | MOL004598 | 3,5,6,7-tetramethoxy-2-(3,4,5-trimethoxyphenyl)chromone | HQ9 | MOL002915 | Salvigenin |
| CH11 | MOL001645 | Linoleyl acetate | HQ10 | MOL002925 | 5,7,2',6'-Tetrahydroxyflavone |
| CH12 | MOL000490 | petunidin | HQ11 | MOL002926 | dihydrooroxylin A |
| CH14 | MOL000422 | kaempferol | HQ12 | MOL002927 | Skullcapflavone II |
| CH15 | MOL000354 | isorhamnetin | HQ13 | MOL002928 | oroxylin a |
| DZ1 | MOL012921 | stepharine | RS1 | MOL005366 | Malvic acid |
| DZ2 | MOL012946 | zizyphus saponin I_qt | RS2 | MOL000942 | (1R,4S,4aR,8aR)-4-isopropyl-1,6-dimethyl-3,4,4a,7,8,8a-hexahydro-2H-naphthalen-1-ol |
| DZ3 | MOL012976 | coumestrol | RS3 | MOL005376 | Panaxadiol |
| DZ4 | MOL012981 | Daechuine S7 | RS4 | MOL001818 | Methyl palmitelaidate |
| DZ5 | MOL012961 | jujuboside A_qt | RS5 | MOL005394 | (Z)-2-methyl-5-[(1S,2R,4R)-2-methyl-3-methylene-2-norbornanyl]pent-2-en-1-ol |
| DZ6 | MOL012986 | Jujubasaponin V_qt | RS6 | MOL001312 | 9-HEXADECENOIC ACID |
| DZ7 | MOL012989 | Jujuboside C_qt | RS7 | MOL005355 | Ginsenoyne E |
| DZ8 | MOL012992 | Mauritine D | RS8 | MOL005272 | 13-Tetradecenyl acetate |
| DZ9 | MOL001454 | berberine | RS10 | MOL005399 | alexandrin_qt |
| DZ10 | MOL001522 | (S)-Coclaurine | RS11 | MOL005294 | 3-methylheptane |
| DZ11 | MOL000211 | Mairin | RS12 | MOL005293 | 3-Ethyl-3-methylheptane |
| DZ13 | MOL003410 | Ziziphin_qt | RS13 | MOL005274 | Neohexane |
| DZ15 | MOL004350 | Ruvoside_qt | RS14 | MOL005381 | 2-Formylpyrrole |
| DZ17 | MOL005360 | malkangunin | RS15 | MOL000749 | Linoleic |
| DZ18 | MOL000627 | Stepholidine | RS16 | MOL001641 | METHYL LINOLEATE |
| DZ19 | MOL007213 | Nuciferin | RS17 | MOL001949 | panaxynol |
| DZ20 | MOL000783 | Protoporphyrin | RS20 | MOL005320 | arachidonate |
| DZ21 | MOL000787 | Fumarine | RS22 | MOL005315 | (R)-()-Citronellal |
| DZ22 | MOL008034 | 21302-79-4 | RS24 | MOL000066 | alloaromadedrene |
| DZ23 | MOL002773 | beta-carotene | RS25 | MOL000935 | Hepanal |
| SJ2 | MOL006129 | 6-methylgingediacetate2 | RS26 | MOL005370 | NN-Dimethyldecanamide |
| SJ4 | MOL001771 | poriferast-5-en-3beta-ol | RS27 | MOL005269 | (+)-Maalioxide |
| SJ5 | MOL008698 | Dihydrocapsaicin | RS28 | MOL005384 | suchilactone |
| FL1 | MOL000273 | (2R)-2-[(3S,5R,10S,13R,14R,16R,17R)-3,16-dihydroxy-4,4,10,13,14-pentamethyl-2,3,5,6,12,15,16,17-octahydro-1H-cyclopenta[a]phenanthren-17-yl]-6-methylhept-5-enoic acid | RS29 | MOL005290 | 3,5-Dimethyl-p-anisic acid |
| FL2 | MOL000275 | trametenolic acid | RS30 | MOL005356 | Girinimbin |
| FL3 | MOL000276 | 7,9(11)-dehydropachymic acid | RS31 | MOL000676 | DBP |
| FL4 | MOL000279 | Cerevisterol | RS32 | MOL003648 | Inermin |
| FL5 | MOL000282 | ergosta-7,22E-dien-3beta-ol | RS33 | MOL005321 | Frutinone A |
| FL6 | MOL000283 | Ergosterol peroxide | RS34 | MOL005308 | Aposiopolamine |
| FL7 | MOL000296 | hederagenin | RS35 | MOL005396 | cis-Widdrol alpha-epoxide |
| FL8 | MOL000289 | pachymic acid | RS36 | MOL002136 | neocnidilide |
| FL9 | MOL000291 | Poricoic acid B | RS37 | MOL004100 | N-Salicylidene-salicylamine |
| FL10 | MOL000287 | 3beta-Hydroxy-24-methylene-8-lanostene-21-oic acid | C1 | MOL000358 | beta-sitosterol |
| FL11 | MOL000290 | Poricoic acid A | D1 | MOL000449 | Stigmasterol |
| FL12 | MOL000292 | poricoic acid C | E1 | MOL000098 | quercetin |
| A1 | MOL002714 | baicalein | G1 | MOL000492 | (+)-catechin |
| B1 | MOL002776 | Baicalin | F1 | MOL000096 | (-)-catechin |
| I1 | MOL002879 | Diop | H1 | MOL000359 | sitosterol |
| H2 | MOL000073 | ent-Epicatechin |  |  |  |

**Note:** BX: Ban Xia, CH: Chai Hu, DZ: Da Zao, FL: Fu Ling, SJ: Sheng Jiang, GZ: Gui Zhi, DH: Da Huang, HQ: Sheng Jiang, RS: Ren Shen, A-H: Common components of different drugs.
